# Management of the patient with allergic and immunological disorders in the pandemic COVID-19 era

**DOI:** 10.1186/s12948-020-00134-5

**Published:** 2020-10-01

**Authors:** Vincenzo Patella, Gabriele Delfino, Giovanni Florio, Giuseppe Spadaro, Fulvia Chieco Bianchi, Gianerico Senna, Mario Di Gioacchino

**Affiliations:** 1 Division Allergy and Clinical Immunology, Department of Medicine, “Santa Maria Della Speranza” Hospital, Salerno, Italy; 2 Postgraduate Program in Allergy and Clinical Immunology, University of Naples Federico II, Naples, Italy; 3 Department of Translational Medical Sciences, University of Naples Federico II, Naples, Italy; 4 Respiratory Diseases Unit, Padua University and General Hospital, Padua, Italy; 5 Asthma Center and Allergy Unit, Verona University and General Hospital, Verona, Italy; 6 Chairman of Italian Society of Allergology, Asthma and Clinical Immunology (SIAAIC), Milan, Italy; 7 Center of Advanced Science and Technology, G. D’Annunzio University, Chieti-Pescara, Italy; 8 Leonardo Da Vinci, University, Chieti, Italy

**Keywords:** COVID-19, Allergy, Asthma, Rhinitis, Autoimmune diseases, Immunodeficiencies, Personal protective equipment

## Abstract

The pandemic COVID-19 abruptly exploded, taking most health professionals around the world unprepared. Italy, the first European country to be hit violently, was forced to activate the lockdown in mid-February 2020. At the time of the spread, a high number of victims were quickly registered, especially in the regions of Northern Italy which have a high rate of highly-polluting production activities. The need to hospitalize the large number of patients with severe forms of COVID-19 led the National Health System to move a large number of specialists from their disciplines to the emergency hospital departments for the treatment of COVID-19. Furthermore, the lockdown itself has limited the possibility for general practitioners and pediatricians to be able to make outpatient visits and/or home care for patients with chronic diseases. Among them, the patient with atopic diseases, such as asthma, rhinitis and atopic dermatitis, is worthy of particular attention as she/he is immersed in a studded negative scenario with the onset of spring, a factor that should not be underestimated for those who suffer from pollen allergy. The Italian Society of Asthma Allergology and Clinical Immunology, to quickly deal with the lack of references and specialist medical procedures, has produced a series of indications for immunologic patient care that are reported in this paper, and can be used as guidelines by specialists of our discipline.

## Background

The pathogenic “Severe Acute Respiratory Syndrome Coronavirus 2” (SARS-CoV-2), after a devastating epidemic in the Chinese Hubei region, with an increase of the epidemic by province, between 20 January and 9 February 2020, with a value of 2.5 (IC 95% 2.4–2.6) [1], spread rapidly all over the world with a particular intensity of contagions and severe cases in the northern regions of Italy [2]. The need to retrieve medical personnel to quickly open COVID-19 departments has reduced the possibility of keeping other specialized healthcare facilities open, such as the Allergology and Clinical Immunology (ACI) Units. Patients with chronic diseases were unable to access their usual specialist centers to receive periodic treatments, and they couldn't even easily consult their family doctors and/or pediatricians because of the lockdown (law decree of 23 February 2020) [3]. Consequently, a real potential risk for the patient stopping the treatment for his allergic or inflammatory diseases during a pandemics with potential risk of infection for emergency health access due to an uncontrolled disease.

Therefore, in addition to the damage directly caused by the infection, there were damages due to the failure to control chronic diseases, often severe, such as immunologic pathologies (autoimmunity, immunodeficiencies, respiratory and skin allergies). Many patients with pollinosis have seen their disease precipitate, coinciding spring, their most disastrous period of the year, with the explosion of the pandemic. The current socio-economic conditions, living habits, the use of the media have strongly influenced, positively and negatively, the spread of this pandemic and the so-called side effects on the medical care of already sick people. Sometimes, these tools have also made it possible to stem the spread of the virus, therefore in this document many initiatives for the control of collateral damage are aimed at promoting, through the media system, remote visits where possible.

### Allergy and clinical immunology unit in the COVID-19 pandemic Italian scenario

**Fig. 1:**
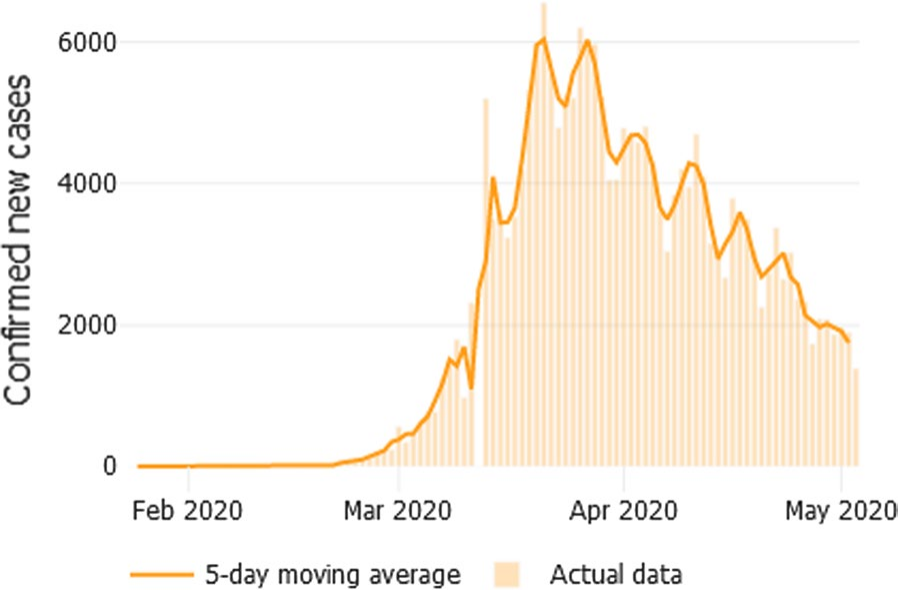
New cases confirmed each day (5-day-average). The first case of COVID-19 in Italy was reported 93 days ago on 31/1/2020. Since then, the country has reported 210,717 cases, and 28,884 deaths [https://coronavirus.jhu.edu/data/new-cases, on 5th of May, 2020]

At the end of February 2020, the first case of severe acute respiratory syndrome due to SARS-CoV-2, that causes COVID-19 disease, was identified in Italy [4]. In the following days, despite the restrictive public health measures aimed at avoiding the spread of the contagion, the number of cases increased. On March 8, 2020, Italy was the 2nd most affected country in the world. The hospitals themselves have on some occasions become a SARS-CoV-2 transmission site, so all normal clinical activities have been discontinued and all specialists have been asked to report a list of medical conditions that need non-deferrable treatment. Some ACI Unit rapidly coordinated a lot of stressful changes in established practices to meet health care needs [5, 6]. The Italian Society of Allergology, Asthma and Clinical Immunology (SIAAIC) has started since March 20, 2020 a series of initiatives (newsletters, webinar [7], Facebook Live with its members and with patient associations) to provide operational recommendations and practical considerations to support doctors involved in the care of patients with allergic and immunologic diseases under critical conditions, i.e. the need to start or continue specialist care under the COVID-19 spectrum: a probable scenario in which the imbalance between supply and demand of specialists for the treatment of these diseases was necessarily determined by the need to find doctors and hospital beds for the increasing number of COVID-19 patients (Fig. 1). So, in the week when the epidemic reached, with over 70,000 cases diagnosed (N = 73,780) and over 6,000 reported deaths (N = 6801) the fastest rate of infection, recommendations were made through a first Webinar with the aim of suggesting to all members non-deferrable medical performances that an ACI Unit should guarantee to patients. In particular the management of severe allergic pathologies (Anaphylaxis, hymenoptera allergy, severe asthma, drug allergy), autoimmune diseases, immunodeficiencies and biological therapies (Box n. 1). Within a few weeks 6 different single-theme Webinars and a document for respiratory allergy patients [8] were licensed. In the following paragraphs some these themes where discussed.

Box 1: List of non-deferrable allergy/immunological procedures

- Management of anaphylactic reactions: to provide patients with (a) prescription of self-injectable adrenaline, (b) indications relating to prevention rules and (c) instructions for the correct self-management of emergency therapy.
- Management of drug allergy: Evaluation and possible allergy tests in patients with suspected drug allergy in case of urgent need for pharmacological treatments or diagnostic-interventional procedures; non-procrastinating procedures for drug desensitization.
- Management of hymenoptera allergy: to ensure continuity of therapy in patients already in treatment with hymenoptera venom immunotherapy; to initiate specific immunotherapy, based on the assessment of patient’s specific risk factors.
- Management of severe pathologies: Clinical evaluation and therapeutic prescriptions for severe asthma; continuation/initiation of off-label treatments in patients with severe allergies.
- Management of immunodeficiencies: intravenous administration of immunoglobulins when subcutaneous route is non practicable.
- Management of autoimmune diseases: ensuring non-deferrable hospital treatment of patients with autoimmune disease.
- Management of biological therapy: ensuring therapeutic continuity in patients being treated with biological drugs that cannot be self-administered.

Box 2: The safe recovery of outpatient activities in the COVID-19 era

- At the official declaration of the cessation of the lockdown phase, in preparing for the resumption of outpatient activities, three phases are distinguished:
  - Phase 1 (pandemic phase, arbitrarily identified by us with an R0 > 1), only specialist outpatient visits, pulse oximetry, blood gas analysis and possible fibro-bronchoscopy are possible.
  - Phase 2 (post-peak phase > 0.5 R0 < 1), in addition to what is already possible in phase 1, simple Spirometry and DLCO, Reversibility test can also be performed (tests possible provided that they are performed with certified filters for high expiratory flows, 600–700 L/min, and provided that environmental sanitization is guaranteed at least twice a day), night-time saturimetry, 6-min walk test, allergy tests.
  - Phase 3 (Complete control of viral infectious disease) All other services are not to be performed until the advent of phase 3 is certified, a phase in which an appropriate defense of the community against the SARS-CoV-2 virus has been achieved [i.e.: skin testing and blood-sample collection, drug provocation tests comprise ENT exams (including endoscopy),bronchoscopy, nasal or bronchial allergen provocation tests, tissue-sampling, lung function tests Pletismography, FeNo measure, oral food challenges and esophageal exams].

### Allergy and clinical immunology and international scientific community activities

As soon as possible the main scientific journals, with basic and clinical Editors announced after Covid-19 Pandemia the possibility of a rapid publication about any papers with this topic. It is interesting report that many National and International Scientific Society followed this invite and a large number of articles are published, in particular in EAACI documents for asthma and COVID-19 is reported that difficult to clearly assess the prevalence of asthma on COVID-19 in many studies since most patients are older adults and probably have multimorbidities. Most studies do not clarify whether asthmatic patients with COVID-19 have isolated asthma or asthma as a multimorbidity, particularly in the context of hypertension, obesity and diabetes. In particular, obesity is a significant risk factor for COVID-19 and its severity, and may be intertwined with asthma [9]. An international Position Paper, recently published by EAACI, provides in nine sections on different relevant aspects for the care of patients with allergies, those recommendations are developed on operational plans and procedures to maintain high standards in the daily clinical care of allergic patients whilst ensuring necessary safety in the current COVID-19 pandemic [10].

On the other hand many groups are heavy working on vaccine solution, but different approaches have been proposed in the world, all of them pending by many months before to have a global vaccine versus COVID-19. Different proposals have been diffused about a immune-solution, one of this hypothesis is could anti-tubercular vaccination protect against covid-19 infection? [11], although, in the absence of evidence it does not recommend BCG vaccination for the prevention of COVID-19 as suggest by WHO [12].

### Patients with asthma rhinitis and conjunctivitis

Allergic patients with manifestation of asthma, rhinitis and conjunctivitis had acute relapses during the COVID-19 emergency both due to the massive spring exposure to allergens and other triggering factors such as internal and external pollutants [10, 13] and to the sudden interruption of current treatment, for fear that the same drugs could facilitate SARS infection. On the contrary, in the current state of knowledge, intranasal corticosteroid therapy for allergic rhinitis can be continued in patients with COVID-19 at the recommended posology [8, 10]. There are no evidences that such therapy can cause immunosuppression and discontinuing treatment for allergic rhinitis can lead to an increase in respiratory symptoms, especially sneezing, with potentially greater spread of the virus. The same recommendation was also suggested for asthmatic patients, the treatment and the dose of inhaled steroids established on the basis of asthma control must be maintained. Patients with asthma have a greater susceptibility to respiratory viral infections which may be a trigger for exacerbations [14], for this reason, pending more reliable evidence, they should still be considered at a high risk of severe COVID-19 outcomes. However, it should be noted, that some authors assume that Th2 polarization does not represent a risk factor. Although higher ACE2 expression increases in vitro susceptibility to SARS-CoV, and studies examining factors that affect ACE2 gene expression have revealed that its upregulation is associated with smoking, diabetes, and hypertension, all of which are associated with increased severity of COVID-19 illness [15]. Some Authors reported that a potential explanation for the unexpected observation that asthma and other allergic diseases may not be a risk factor for severe COVID-19 disease is a reduced ACE2 gene expression in airway cells and thus decreased susceptibility to infection. To test this hypothesis, they examined whether asthma and respiratory allergy are associated with reduced ACE2 expression in airway cells from 3 different cohorts of children and adults and they concluded that: respiratory allergy and controlled allergen exposures are each associated with significant reductions in ACE2 expression. ACE2 expression was lowest in those with both high levels of allergic sensitization and asthma. Importantly, no atopic asthma was not associated with reduced ACE2 expression [16]. It could be speculated that Th2-dominant environment can be also protective, able to down-regulate the late phase hyper-inflammation which typically marks severe respiratory viral diseases, when the viral load decreases but immunopathologic events are the hallmarks of tissue damage [17]. In this regard, it could be very interesting and useful to check COVID-19 incidence and manifestations in asthmatic patients’ network. Despite, any asthma exacerbations had to be immediately communicated by phone to the specialist, who would evaluate after a short triage the possible therapeutic changes to be adopted. Asthmatics should adopt strict protection measures and the CDC statement should be used [9]. All recommendation should be updated regularly in light of the continuous acquisitions on COVID-19 [10, 18]. Specialists are recommended to suggest to patients an emergency therapeutic protocol to be implemented in case of asthmatic exacerbation, and to give the possibility of direct telephone contacts, also to manage possible psychological tensions or real panic attacks in the doubt of having contracted the SARS-CoV-2 infection.

### Patients being treated with biological drugs

Particular attention goes to patients who, due to the severity of the disease, must add biologics to their conventional therapy, because some of them are administered in hospital setting and in any case the management of the treatment (even when the biologic can be self-administered at home) should be supervised by a trained specialist. The recommendations shared by SIAAIC experts foresee: close monitoring and a therapeutic attitude that must be assessed case by case by the clinician for a house therapy; telephone consultations are highly recommended for all patients; do not stop or change the schedule of the treatment without consulting the specialist at the ACI Unit. ACI Units expert in biological treatment of severe asthma, urticaria and atopic dermatitis are present in each region. Specialists are highly recommended to consider switching to the subcutaneous self-administration all possible biologics. In particular, administration of Dupilumab can be done at home after the first administration in the ACI Unit [19]; Omalizumab can be administered at home from the fourth administration in absence of history of anaphylaxis; Benralizumab home administration should be evaluated after training the patients to self-administration, on the contrary, Mepolizumab should be administered by the doctor at the ACI Unit [20].

### Allergen immunotherapy

Allergen-specific immunotherapy (AIT) is the causal treatment of allergic conditions and is effective in reducing symptoms and therapeutic load in allergic rhinitis and asthma. In Venom allergy is considered a life-saving treatment [10]. Giving the limited experimental data so far it does not seem that suppression of Th2 Cell and the induction of a regulatory B and T response, which are clearly antigen specific, could interfere with the immune response to the SARS-CoV-2 virus. Previous data on influenza, CMV and HIV infected patient have indeed demonstrated that AIT was safe and well tolerated. Therefore in non infected individuals during COVID-19 pandemics or in those who completely recovered after the infection interruption of sublingual immunotherapy (usually taken at home) is not advised. Specialists should recommend their patients to have sufficient supply of medication. Conversely, in COVID-19 diagnosed patients or suspected (close contact to SARS-CoV-2 positive individuals) AIT should be interrupted [21]. Subcutaneous immunotherapy can be continued under strict safety protocols considering injection intervals expansion especially in hymenoptera Venom allergy, a potentially life-threatening condition. Ongoing mite and pollen AIT can help reducing allergic symptoms such as sneezing or coughing. Allergy service staff should follow all recommended measures for infection prevention and control for droplet, contact and airborne transmission. Interrupting all subcutaneous immunotherapies is advised in COVID-19 diagnosed patients.

### Patients being treated with immunosuppressive and biologic drugs

Patients with autoimmune diseases who take immunosuppressant drugs should be recommended to proceed with chronic therapy. It is essential that these patients would scrupulously follow all the preventive measures suggested for COVID-19, in order to avoid infections in the unfortunate case of contagion with immunosuppressants [22]. Other drugs for the treatment of autoimmune diseases should be continue also in case of SARS-CoV-2 contagion. In fact, such drugs are potentially useful in the management of COVID-19. The cytokine storm observed in acute respiratory distress syndrome induced by the severe SARS-CoV-2 infection can be, almost partially, controlled by anti-IL-1 and anti-IL-6. Baricitinib has antiviral and anti-inflammatory properties. Similar consideration can be done for some anticancer drugs, considering that immunotherapy with immune checkpoint inhibitors is able to restore the cellular immunocompetence, as suggested in the context of influenza infection, the patient undergoing immune checkpoint blockade could be more immunocompetent [23]. Ongoing trials definitively clarify these aspects.

### Patients with immunodeficiency being treated with immunoglobulins

It is recommended for these patients to continue with immunoglobulin replacement therapy, preferring the subcutaneous (home) route and carry out a case-by-case clinical evaluation, also using telephone triage, to identify the need for hospitalization in case of deterioration of the overall clinical picture. In subjects unable to perform subcutaneous therapy, intravenous immunoglobulin administration must be continued at the ACI Units, scrupulously implementing all preventive measures for SARS-CoV-2 contagion. Among patients with humoral immunity deficiency [X-linked Agammaglobulinemia (XLA), Autosomal Recessive Agammaglobulinemia (ARA), Common Variable Immunodeficiency (CVID)], those with Agammaglobulinemia (XLA and ARA) are sensitive to a limited number of viral infections, mainly norovirus, enterovirus and poliovirus with increased incidence of post vaccination polio oral attenuation of Sabin, while those with CVID are more susceptible to rhinovirus, norovirus and herpesvirus. When affected by COVID-19, patients with Agammaglobulinemia showed a more favorable course than patients with CVID especially for the more severe forms, thus offering food for thought on the putative mechanisms underlying the immunological response to the infection and suggesting possible indications for new therapeutic targets [24].

### Allergy risk for health workers wearing personal devices

Clear communication of guidelines on protection against COVID-19 infections is considered vital. Unfortunately, in some cases, the lack and/or poor-quality of personal protective equipment (PPE) was a serious concern for health workers and managers and the reason for the spreading of COVID-19 among health personnel. Furthermore, in a recent metanalysis of 36 studies including 20 sampled studies (ten from Asia, four from Africa, four from Central and North America and two from Australia) which gathered the views and experiences of nurses, doctors and other health professionals when dealing with severe acute respiratory syndrome (SARS), H1N1, Middle Eastern respiratory syndrome (MERS), tuberculosis (TB) or seasonal flu [25], that there is a lack of training on infection itself and how to use the PPE. The protection protocol from contagion must include, among other procedures, protecting operators with gowns, hats, gloves disposable socks. Many of these PPE can induce allergic or irritant contact dermatitis, therefore it is essential informing and training health personnel to the use of PPE. Wearing the mask for a long time may cause a severe irritation of the skin of the face and sometimes real injuries. Prevention can be obtained applying a light layer of hypoallergenic cream on the face in order to create a first "protective barrier" between the mask and the skin. Often nasal masks possess a flexible metal insert, to ensure a better adhesion to the face; such an insert can cause pressure injury or irritant/allergic contact dermatitis [26, 27]. Applying a soft layer of fabric or a rubber patch between the insert in metal and the skin, or along the entire edge of contact between the mask and the face, can prevent the appearance of the lesions. Soothing and anti-inflammatory creams are useful to treat such irritation and injuries; topical antibiotic and healing products are indicated in the presence of ulcerations. Finally, the use of alcohol-based hand rubs should be encouraged in healthcare professionals, and, as they are at risk of developing occupational dermatitis, skin cleansers with weak allergens must be used and gloves without accelerators (thiazoles, thiurams and carbamates) and latex-free are recommended [28, 29].

## Conclusions

The sudden expansion of the COVID-19 pandemic has found many doctors unprepared and has caused difficulties in treating chronic diseases for most patients. In this event drastic measures may be needed, and limit or require adjustment of ambulatory allergy services. However, no rationale for how to prioritize service shut down and patient care exists. During the ongoing pandemic while social distancing is being encouraged, most allergy/immunology care could be postponed/delayed or handled through virtual care [30]. With the exception of many patients with primary immunodeficiency, patients on venom immunotherapy, and patients with asthma of a certain severity, there is limited need for face-to-face visits under such conditions [20, 29]. The Italian Society of Allergology, Asthma and Clinical Immunology quickly shared references and procedures for patients suffering from allergic and/or immunological diseases within members. Such operative procedures can be useful for all doctors who can find more information on the society website at https://www.siaaic.org and ARIA-EAACI statement on Asthma and COVID-19.

## Data Availability

All materials and data are available to publication.
